# Combined analysis of sMRI and fMRI imaging data provides accurate disease markers for hearing impairment^☆^

**DOI:** 10.1016/j.nicl.2013.09.008

**Published:** 2013-10-11

**Authors:** Lirong Tan, Ye Chen, Thomas C. Maloney, Marguerite M. Caré, Scott K. Holland, Long J. Lu

**Affiliations:** a Division of Biomedical Informatics, Cincinnati Children's Hospital Research Foundation, 3333 Burnet Avenue, Cincinnati, OH 45229-3026, USA; b School of Computing Sciences and Informatics, University of Cincinnati, 810 Old Chemistry, Cincinnati, OH 45221-0008, USA; c School of Electronics and Computing Systems, University of Cincinnati, 497 Rhodes Hall, Cincinnati, OH 45221, USA; d Pediatric Neuroimaging Research Consortium, Cincinnati Children's Hospital Medical Center, Cincinnati, OH 45221, USA; e Department of Pediatric Radiology, Cincinnati Children's Hospital Medical Center, Cincinnati, OH 45221, USA; f Department of Environmental Health, College of Medicine, University of Cincinnati, 231 Albert Sabin Way, Cincinnati, OH 45267-0524, USA

**Keywords:** Brain, Hearing loss, MRI images, sMRI, fMRI, SVM

## Abstract

In this research, we developed a robust two-layer classifier that can accurately classify normal hearing (NH) from hearing impaired (HI) infants with congenital sensori-neural hearing loss (SNHL) based on their Magnetic Resonance (MR) images. Unlike traditional methods that examine the intensity of each single voxel, we extracted high-level features to characterize the structural MR images (sMRI) and functional MR images (fMRI). The Scale Invariant Feature Transform (SIFT) algorithm was employed to detect and describe the local features in sMRI. For fMRI, we constructed contrast maps and detected the most activated/de-activated regions in each individual. Based on those salient regions occurring across individuals, the bag-of-words strategy was introduced to vectorize the contrast maps. We then used a two-layer model to integrate these two types of features together. With the leave-one-out cross-validation approach, this integrated model achieved an AUC score of 0.90. Additionally, our algorithm highlighted several important brain regions that differentiated between NH and HI children. Some of these regions, e.g. planum temporale and angular gyrus, were well known auditory and visual language association regions. Others, e.g. the anterior cingulate cortex (ACC), were not necessarily expected to play a role in differentiating HI from NH children and provided a new understanding of brain function and of the disorder itself. These important brain regions provided clues about neuroimaging markers that may be relevant to the future use of functional neuroimaging to guide predictions about speech and language outcomes in HI infants who receive a cochlear implant. This type of prognostic information could be extremely useful and is currently not available to clinicians by any other means.

## Highlights

• We probe brain structural and functional changes in hearing impaired (HI) infants.
• We build a robust two-layer classifier that integrates sMRI and fMRI data.
• This integrated model accurately separates HI from normal infants (AUC 0.9).
• Our method detects important brain regions different between HI and normal infants.
• Our method can include diverse types of data and be applied to other diseases.

## 1 Introduction

It has been estimated that approximately 1 to 6 infants per 1000 are born with severe to profound congenital sensori-neural hearing loss (SNHL) (Bachmann and Arvedson, 1998; Cunningham and Cox, 2003; Kemper and Downs, 2000; Northern, 1994). Those children receive little or no benefit from hearing aids and face challenges in developing language abilities due to their inability to detect acoustic–phonetic signals, which are essential for hearing-dependent learning. Cochlear implantation (CI) is a surgical procedure that inserts an electronic device into the cochlea for direct stimulation of the auditory nerve and has been demonstrated to be effective in restoring hearing in patients suffering from SNHL. Statistical data from the National Institute on Deafness and Other Communication Disorders (NIDCD) indicate that approximately 28,400 children in the United States have received a cochlear implant as of December 2010. While many congenitally deaf CI recipients achieve a high degree of accuracy in speech perception and develop near-normal language skills, about 30% of the recipients do not derive any benefit from the CI (Niparko et al., 2010). A deeper understanding of hearing loss and better characterization of the brain regions affected by hearing loss will help reduce the high variance in CI outcomes and result in a more effective treatment of children with hearing loss.

In recent years, Magnetic Resonance (MR) images have been used to study neurological disorders and brain development in children, such as reading and attention problems, traumatic brain injury, hearing impairment, perinatal stroke and other conditions (Horowitz-Kraus and Holland, 2012; Leach and Holland, 2010; Smith et al., 2011; Tillema et al., 2008; Tlustos et al., 2011). Brain MRI scans have revealed significant differences between Hearing Impaired (HI) and Normal Hearing (NH) children. Jonas et al. reviewed a total number of 162 patients' structural MRI scans, and detected 51 abnormalities in 49 patients. Those abnormalities included white matter changes, structural or anatomical abnormalities, neoplasms, gray matter changes, vasculitis and neuro-metabolic changes (Jonas et al., 2012). Similar studies have showed consistent results (Lapointe et al., 2006; Smith et al., 2011; Trimble et al., 2007). Furthermore, functional MRI studies have demonstrated that the activation pattern of HI is different from that of NH during certain scanning tasks (Bilecen et al., 2000; Patel et al., 2007; Propst et al., 2010; Scheffler et al., 1998; Tschopp et al., 2000). For example, Propst and colleagues studied the activation pattern of HI with narrowband noise and speech-in-noise tasks (Propst et al., 2010). In the narrowband noise task, they found that HI children had weaker activation in the auditory areas when compared to NH children. Meanwhile, NH also activated auditory association areas and attention networks, which were not detected in HI children. In the speech-in-noise task, HI children activated the secondary auditory processing areas only in the left hemisphere, rather than bilaterally as is typical of NH. Recently, we have tried to use the activation in the primary auditory cortex (A1) to predict CI outcomes. A strong correlation (linear regression coefficient, R = 0.88) was detected between the improvement in post-CI hearing threshold and the amount of activation in the A1 region before CI (Patel et al., 2007). Despite these recent advances, it remains unclear whether these structural and functional abnormalities are sufficient to distinguish HI from NH individuals.

In this study, we set out to investigate whether we can accurately classify HI from NH individuals based on MR images alone by utilizing machine learning techniques. We have trained three classifiers, one based on structural MR (sMRI) images, another based on functional MR (fMRI) images, and a third that integrates sMRI and fMRI images. While traditional methods utilize voxel-based morphometric (VBM) features, in which each single voxel serves as an independent feature, we extracted high-level features to characterize the 3D images. Specifically, we employed the Scale Invariant Feature Transform (SIFT) algorithm to detect and describe local features in sMRI and extracted region-level features to represent the functional contrast maps. Based upon the extracted features, SVM classifiers were trained to separate HI from NH.

The SIFT algorithm was first proposed by Lowe for object recognition (Lowe, 1999). Since then, it has been widely used in the computer vision field. Basically, the SIFT algorithm detects blob-like image components and calculates a vector to describe each of these components. Each vector becomes a SIFT feature. The set of SIFT features extracted from an image contains important characteristics of this image and can be used for subsequent analysis, e.g. object recognition, gesture recognition etc. In this study, we employed the SIFT algorithm to extract SIFT features from brain structural MR images, and devised an approach for the automatic classification of NH vs. HI based on the SIFT features.

There are three levels of significance for this study. First of all, we convincingly demonstrate that hearing loss can be accurately diagnosed based on MR images alone. Secondly, brain regions identified by the classifiers enable us to better understand hearing loss, and may serve as valuable indicators for the CI outcome and facilitate follow-up treatment post-CI (Jonas et al., 2012). Finally, our algorithm can be easily extended to assist in diagnosing other disorders affecting children's brains, e.g., speech sound disorders of childhood, leading to a path for improving child health.

The organization of this article is as follows. In Materials and methods, we describe in sequence the data sources and the preprocessing procedures, the methods of analyzing sMRI and fMRI images, the integrative model that combines these two methods, and the validation of our classifiers. In Results, we compare the classification performance of the sMRI classifier, the fMRI classifier and the combined classifier, and assess the stability of feature selection as well as the discriminatory power of features. Finally, in Discussion, we summarize the present work, highlight the significance of our approach, and discuss the limitations and envisioned future improvements. We also examine the predictive brain regions our classifiers identified and discuss their relevance in the context of hearing loss.

## 2 Materials and methods

### 2.1 Data acquisition and preprocessing

#### 2.1.1 Participants

Thirty-nine infants and toddlers participated in a clinically indicated MRI brain study under sedation. This study was conducted with approval from the Cincinnati Children's Hospital Medical Center Institutional Review Board (IRB). Eighteen of the participants had SNHL (10 females, average age = 14 months, range = 8–24 months). All hearing impaired participants were referred by the Division of Otolaryngology for MRI as part of the cochlear implant staging process and consented to participate in our adjoining fMRI protocol. They had documented bilateral severe to profound hearing loss with average hearing thresholds in the range of 90 dB or greater. Nine of these subjects had no measureable hearing response in either ear at the maximum level of our audiometry equipment, at 120 dB and can be considered deaf. The remaining 21 participants were normal hearing controls (15 females, average age = 12 months, range = 8–17 months). These children received clinical MRI scans with sedation for non-hearing related indications. They were recruited for the control group if they met the inclusion criteria: gestational age of at least 36 weeks, normal otoacoustic emissions hearing, and normal neuroanatomy determined by the neuroradiologist. Informed consent of parent or guardian was obtained prior to the study protocol, and the parent agreed to additional hearing tests at a separate visit. The child's reason for referral for brain MRI was not related to hearing. Exclusions included head circumference < 5 percentile or > 95 percentile, orthodontic or metallic implants that interfere with the MRI, abnormal brain pathology in the central auditory pathways. Examples of indications for scanning in this group were, “odd body positioning-rule out chiari malformation”, “recent onset irritable behavior-rule out brain tumor”. All participants were screened for hearing loss using otoacoustic emission (OAE) prior to the MRI scan. Failed OAE at the time of scan was also an exclusion criterion for the normal control group. All of these brain scans of both hearing impaired group and control group were reviewed by a pediatric neuroradiologist and assessed as having no anatomical findings of significance. One of the challenges of research in pediatric neuroimaging is that it is unethical to expose children to more than minimal risk for the purposes of research. This principle is dictated by our conscience as well as by the IRB at most institutions. Consequently, one of the fine points in the design of the present study is that we were required to select our control population among infants who were referred for an MRI scan with sedation because of a clinical indication. With the precautions described above and other procedures we took to insure normal auditory function and brain anatomy, this is perhaps the best control group that could be obtained for this age group in an ethical fashion. However, it is important to note that the controls were not randomly sampled from the general population.

#### 2.1.2 MRI/fMRI acquisition

Anatomical images for this study were acquired using a 3.0 Tesla Siemens Trio MRI scanner in the clinical Department of Radiology. Isotropic images of the brain were acquired using an inversion recovery prepared rapid gradient-echo 3D method (MP-RAGE) covering the entire brain at a spatial resolution of 1 × 1 × 1 mm in an axial orientation. 3D MP-RAGE acquisition parameters were as follows: TI/TR/TE = 1100/1900/4.1 ms, FOV = 25.6 × 20.8 cm, matrix = 256 × 208, scan time = 3 min and 50 s. These high resolution 3D-T1 weighted images were used for co-registration of fMRI scans which were also acquired during this scheduled MRI.

**Fig. 1:**
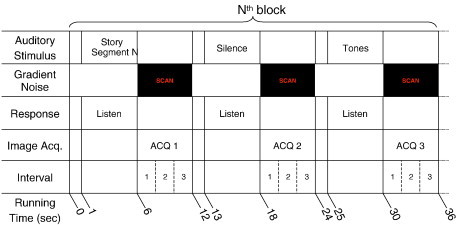
Timing diagram of a single block of the HUSH fMRI data acquisition and auditory stimulation paradigm. MRI scans and accompanying gradient noise occurred in the intervals labeled “scan”. During other intervals of the sequence, the scanner remained silent. Sound level inside the scanner was 104 dB during the acquisitions and ~ 60 dB during the silent intervals. This block was repeated 18 times for a total scan duration of approximately 11 min.

Functional MRI scans were performed using a silent background fMRI acquisition technique that allowed auditory stimuli to be presented during a silent gradient interval of the scan, followed by an acquisition interval that captured the peak BOLD response of relevant brain regions (Schmithorst and Holland, 2004). Using the scanner described above we acquired BOLD fMRI scans in an axial plane (4 × 4 mm resolution), using the manufacturer's standard gradient echo, EPI sequence covering the same FOV as the 3D T1 images (see paragraph above), with the following parameters: TR/TE = 2000/23 msec, flip angle = 90°, matrix = 64 × 64 and 25 axial slices with thickness = 5 mm. In the present study, all stimulus and control intervals were of equal duration (5 s) in a three-phase auditory paradigm consisting of speech, silence, and narrow band noise tones interleaved with acquisition periods of 6 s during which 3 image volumes were obtained covering the whole brain. A timing diagram for the fMRI data acquisition and stimulation paradigm is shown in Fig. 1.

The speech stimulus consisted of sentences read in a female voice. Altogether 36 sentences were read in 18 segments of 5 s duration and comprising 2 sentences each. This condition was followed by a 6 s data acquisition and then a 5 s interval of silence as a control condition. After another 6 s control interval acquisition, a second auditory control condition was played. This condition consisted of Narrow Band Noise (NBN) tones patterned after standard audiology evaluations for detection of hearing thresholds. Five NBN tones of 1 s duration with center frequencies of 250, 500, 1000, 2000 and 4000 Hz and bandwidth of 50% were played in random order during this control condition, for a total of 5 s during a silent interval of the scanner. An additional interval of 1 s of silence followed each acquisition to provide an acoustic demarcation prior to the stimulus onset of each stimulus condition. This resulted in the fMRI acquisition time of approximately 11 min. See Fig. 1 for a detailed schematic of the task and timing. Auditory stimuli were administered through calibrated MR compatible headphones at a sound level of 10–15 dB greater than the individual participant's Pure Tone Average (PTA) hearing threshold. Each hearing impaired participant in the study had a recent audiogram, which was used to determine the sound level for fMRI. Our MR compatible audio system was modified to allow for an output through the headphones measuring up to 130 dB.

#### 2.1.3 Data analysis — preprocessing

fMRI data were initially analyzed on a voxel-by-voxel basis to identify the activated brain regions using a standard pre-processing pipeline implemented in the Cincinnati Children's Hospital Image Processing Software (CCHIPS) (Schmithorst et al., 2010) written in IDL computer language. In this paper, we use voxel for 3-dimensional images and pixel for 2-dimensional images.

Since the subjects were sedated, we assumed that the anatomical image was naturally aligned with the functional images for each individual. Therefore, alignments between anatomical images and functional images were not needed in preprocessing. In this case, it does not matter if we apply the normalization transformation before or after contrast determination. To generate both normalized contrast maps used in the current study as well as contrast maps in native space for other uses, we first generated contrast maps in each individual's native space and then normalized the contrast maps to standard space. The raw EPI images were simultaneously corrected for Nyquist ghosting and geometrical distortion (due to B0 field inhomogeneity) (Schmithorst et al., 2001). EPI functional MR time-series images were corrected on a voxel-by-voxel basis for drift using a quadratic baseline correction. Motion artifacts were corrected using a pyramid iterative co-registration algorithm (Thevenaz et al., 1998). During this stage, infant brain images were transformed to the AC–PC plane. Finally, the individual image volumes (1,2,3) in the event-related fMRI acquisition were separated and submitted to a final pre-processing step using the General Linear Model (Worsley et al., 2002) to construct individual Z-maps for each volume and contrast condition (speech vs. silence, speech vs. tones and tones vs. silence). Z-maps showing activation for each condition for each participant were then computed by averaging the Z-maps from the individual volumes for each contrast condition (Patel et al., 2007; Schmithorst and Holland, 2004). These Z-maps, in each individual's native space were used by the radiologists and neurotologists for clinical interpretation of findings. The neuroradiologist reviewed both functional and anatomical MRI scans for each participant and completed a standardized report indicating whether brain abnormalities or brain activities were detected in primary auditory areas, language areas or other brain regions. After that, we performed spatial normalization using SPM8 with a T1 template constructed from a control group of age matched subjects selected specifically for this infant cohort (Altaye et al., 2008). The normalized anatomical images and functional Z-maps were then submitted to the next stages of analysis.

### 2.2 Feature extraction and model learning based on structural MR images

For sMRI images, we used SIFT features to represent the brain images and developed an algorithm to analyze the SIFT features. We have previously applied this method to Alzheimer's disease, Parkinson's disease and bipolar disease, and it has demonstrated promising classification performance (Chen et al., 2013).

#### 2.2.1 Obtaining 2D slices from 3D brain images

Due to the high density of SIFT features in the brain images and the pair-wise comparison among SIFT features required in a later step, analyzing the 3D brain image as a whole is computationally infeasible. Thus, the spatially normalized 3D brain (157 × 189 × 136) was divided into 560 20 × 20 × 20 cubes. Since the dimensions of brain image were not divisible by 20, the cubes at the end of dimensions only contained the remaining volume of the brain image and therefore had a size smaller than 20 × 20 × 20. The number 20 was determined based on our experience from the application of this algorithm to several other diseases. The cube size mainly affects the computation speed and accuracy of the likelihood scores as described in the Feature evaluation section below. A larger size leads to a much longer computation time, while a smaller size decreases the accuracy of likelihood scores and subsequently leads to lower classification accuracy. According to our experimental results, the cube size 20 × 20 × 20 provides a good balance between speed and accuracy. Every cube was sliced along three different orientations to obtain 3 sets of 20 2D brain images. We analyzed every cube and every set of 2D brain images individually. The analysis results were combined together in the last step.

#### 2.2.2 Extracting SIFT features

**Fig. 2:**
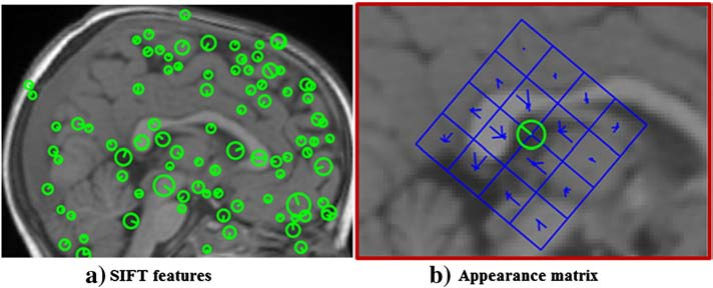
SIFT features. (a) SIFT features extracted from a 2D slice of a brain MR image. Every circle represents a feature. The center of a circle represents the location of the feature. The radius of the circle represents the scale of the feature. There is an appearance matrix associated with every SIFT feature. (b) An appearance matrix consists of a 4 × 4 squares. Every square contains an orientation histogram to represent the gradient orientations of the pixels within this square (Lowe, 1999). The length of the short lines within the squares represents the number of pixels with the corresponding gradient orientation.

The SIFT algorithm for analyzing 2D images was implemented in several stable software packages (Lowe; Vedaldi and Fulkerson, 2010). In this study, we used the SIFT algorithm provided in a publicly available computer vision software package vlFeat (Vedaldi and Fulkerson, 2010). The SIFT features are described by center locations, scales, orientations and appearance matrices. An example of SIFT features is shown in Fig. 2. The SIFT features are shown as circles in Fig. 2(a). Each circle represents a SIFT feature. The center and radius of the circle represent the center location and the scale of the SIFT feature. The existence of a SIFT feature suggests that there is a blob-like image component at the center location of the SIFT feature and the scale of the feature represents the radius of the blob-like component. The image intensity distribution around the blob-like component is further characterized by an orientation and an appearance matrix. The orientation, as shown by the line starting from the center of the circle, represents the general direction of change in image intensity. The appearance matrix represents the detailed change in image intensity. An example of an appearance matrix is shown in Fig. 2(b). The square centered at the center location of a SIFT feature is divided into 16 subsquares. There are 8 lines starting from the center of each subsquare along 8 different directions. The length of a line represents the number of pixels which have a gradient direction the same as the line, and some of the lines may have a length of zero. For example, many of the pixels in the lower left corner subsquare, as shown in Fig. 2(b), have a gradient direction pointing to the lower side of the image; therefore the length of the line starting from the center of this subsquare and pointing to the lower side is long. The center location, scale, direction and appearance matrix of a SIFT feature can be organized as a vector of 133 numbers: the center location includes 3 numbers representing its coordinates in the 3D volume of the brain image; the scale and orientation is represented as one number respectively; the appearance matrix is represented by 128 numbers, 8 numbers for each of the 16 subsquares. This vector form is used in the computation; while the isomorphic graph representation, as shown in Fig. 2, is used as a user friendly way of representing the SIFT features.

#### 2.2.3 Feature evaluation

The extracted SIFT features were identified as one of the three feature types, namely patient feature, healthy feature and noise feature. The features were evaluated based on their frequencies of occurrence in patient brains and healthy brains.

There were two steps to evaluate the features, and each SIFT feature was evaluated separately. The first step was to find all the other features that were similar to the feature that was being analyzed. The similarity between two features was measured by four criteria: the distance between the center locations Δ*_x_*(*i*, *j*), the scale difference Δ*_σ_*(*i*, *j*), the orientation difference Δ*_o_*(*i*, *j*) and the difference between their appearance matrix Δ*_a_*(*i*, *j*). They were defined as follows:

(1) $$\Delta_{x}\left(i,j\right)=\frac{{\left\|x_{i}-x_{j}\right\|}_{2}}{\sigma_{i}}$$

(2) $$\Delta_{\sigma }\left(i,j\right)=\left|\ln\frac{\sigma_{j}}{\sigma_{i}}\right|$$

(3) $$\Delta_{o}\left(i,j\right)=\min\left(\left|o_{i}-o_{j}\right|,2\pi -\left|o_{i}-o_{j}\right|\right)$$

(4) $$\Delta_{a}\left(i,j\right)={\left\|a_{i}-a_{j}\right\|}_{2}$$

where *x_i_* was the center location of feature *i*, *σ*_*i*_ was the scale of feature *i*, *o_i_* was the orientation angle of feature *i* and *a_i_* was the appearance matrix of feature *i*. If all the four differences were less than their corresponding threshold, two features were considered to be similar. All the features that were similar to feature *i* constituted the similar feature set for feature *i*:

(5) $$S_{i}=\left\{f_{j}:\Delta_{x}\left(i,j\right)<\epsilon_{x}\wedge \Delta_{\sigma }\left(i,j\right)<\epsilon_{\sigma }\wedge \Delta_{o}\left(i,j\right)<\epsilon_{o}\wedge \Delta_{a}\left(i,j\right)<\epsilon_{a}\;\right\}$$

where *ϵ*_*x*_, *ϵ*_*σ*_, *ϵ*_*o*_ and *ϵ*_*a*_ were similarity thresholds for center locations, scales, orientations and appearance matrix, respectively. According to Toews et al. (2010), the thresholds *ϵ*_*x*_ and *ϵ*_*σ*_ were set to 0.5 and 2/3 respectively. The thresholds *ϵ*_*o*_ and *ϵ*_*a*_ were set to *π*/2 and 0.45 respectively based on a grid search (Chang and Lin, 2011). Grid search is an efficient way to find the best parameter combinations, when there are multiple parameters in a model and the parameters are continuous variables. First, we discretized the continuous parameters. Parameter *ϵ*_*o*_ was discretized into three discrete values [*π*/4, 2*π*/4, 3*π*/4], and parameter *ϵ*_*a*_ was discretized into five discrete values [0.3, 0.35, 0.4, 0.45, 0.5]. Then all the combinations of these discrete values, 15 combinations in total, were tried and the parameter combination with the highest classification accuracy was chosen as the best parameter setting.

The second step for feature evaluation was to assign likelihood scores to the SIFT features. The likelihood score was defined as follows:

(6) $$L_{i}=\left\{\begin{array}{cc} \ln\frac{\left|S_{i}\cap P\right|/N_{P}}{\left|S_{i}\cap C\right|/N_{C}}, & \left|S_{i}\right|\geq N_{P}+N_{C}\\ 0 & \;\mathrm{otherwise} \end{array}\right)$$

where *S_i_* was the similar feature set for SIFT feature *i*, P was the patient feature set which included all the SIFT features extracted from all patient brains in the training set, C was the healthy feature set including all the SIFT features from all healthy brains in the training set, *N_P_* and *N_C_* was the number of patient brains and the number of healthy control brains in the training set, respectively.

A SIFT feature was identified as a patient feature if *L_i_* was larger than a threshold *ϵ*_*l*_; it was a healthy feature if *L_i_* was smaller than − *ϵ*_*l*_; it was a noise feature otherwise. Formally, the class labels of the features were determined as follows:

(7) $$C_{i}=\left\{\begin{array}{cc} 1, & L_{i}>\epsilon_{l}\\ 0, & \left|L_{i}\right|\leq \epsilon_{l}\\ -1, & L_{i}<-\epsilon_{l} \end{array}\right)$$

where *ϵ*_*l*_ was the threshold for likelihood scores. We used grid search to determine the best parameter setting. For the threshold, the value from 0.1 to 1.2 with a step size of 0.1 was searched. After the grid search, *ϵ*_*l*_ was set to be 0.9.

According to the above feature evaluation process, we need to find the similar feature set for every feature (Eq. (5)), which requires comparing this feature with all other features. For more than 10^5^ features in 39 brains, it would require 10^10^ pair-wise distance calculations, which is a very slow process. Upon those observations, we divided the whole brain volume into small cubes. For the evaluation of a feature, we only calculated its distance to the other features in the same cube. In this way, the computation time is significantly reduced, but the classification accuracy may be adversely affected. For example, a feature close to cube boundaries may have some of its similar features (Eq. (5)) in adjacent cubes. Ignoring those similar features in adjacent cubes could lead to an inaccurate likelihood score (Eq. (6)) for this feature. This issue is especially serious when the number of training samples is limited as in our project. On the other hand, a larger cube size would have fewer features close to cube boundaries, and would result in more accurate likelihood scores and hence higher classification accuracy. According to our previous experience from the application of this algorithm to the classification of several other diseases, such as Parkinson's disease, Alzheimer's disease and bipolar disorder, 20 × 20 × 20 was considered to be an appropriate cube size. This cube size 20 × 20 × 20, determined based on adult-sized brains in our previous studies, was used directly for the infant brains in the present study, since our infant brains were normalized using the infant template and the infant template was enlarged to the size very close to that of adult brains (Altaye et al., 2008).

#### 2.2.4 Training SVM classifiers

We trained a linear SVM for every set of 2D slices in every cube to classify the set of SIFT features extracted from this set of 2D slices across subjects into 3 categories. For a new SIFT feature from a brain image whose class-label is unknown, the corresponding SVM is expected to be able to predict the class label of this new SIFT feature without finding its similar feature set in the huge amount of SIFT features extracted from the brain images used for training.

#### 2.2.5 Predicting new subjects

To predict a new subject to be NH or HI, the subject's sMRI scan was first normalized to the standard space using SPM8 with the infant T1 template (Altaye et al., 2008). The normalized brain was divided into cubes and sliced along three orientations as described above. SIFT features were extracted and then classified using the SVM that was trained for the same cube and same slice orientation. After all the SIFT features were classified, we counted the number of features of the three types. The total number of noise features was not used in the final decision process. The new subject was classified according to the following equation:

(8) $$\mathrm{Class}\;\mathrm{label}=\left\{\begin{array}{cc} \mathrm{HI} & ,\mathrm{if}\;C_{\mathrm{sum}}>\epsilon_{s}\\ \mathrm{NH} & ,\mathrm{otherwise} \end{array}\right)$$

where $C_{\mathrm{sum}}=\sum_{i}{\hat{C}}_{i}$, ${\hat{C}}_{i}$ is the predicted class label of the *i*-th SIFT feature as shown in Eq. (7), *ϵ*_*s*_ is a threshold for the final classification of sMRI and its value is determined based on the method described in section Validation of the classifier.

### 2.3 Feature extraction and model learning based on functional MR images

For fMRI images, we constructed contrast maps using the General Linear Model (GLM) (Worsley et al., 2002) as described in the Data acquisition and preprocessing section. Contrast values were estimated from the difference in image intensity for each voxel between two conditions. A positive contrast value indicated that brain activation was higher in the first condition when compared to the second condition, while a negative contrast value suggested a lower activation in the first condition. We generated region-level features and proposed a novel approach to vectorize the contrast maps utilizing the “bag-of-words” strategy (Sivic and Zisserman, 2009).

#### 2.3.1 Feature generation from contrast maps

**Fig. 3:**
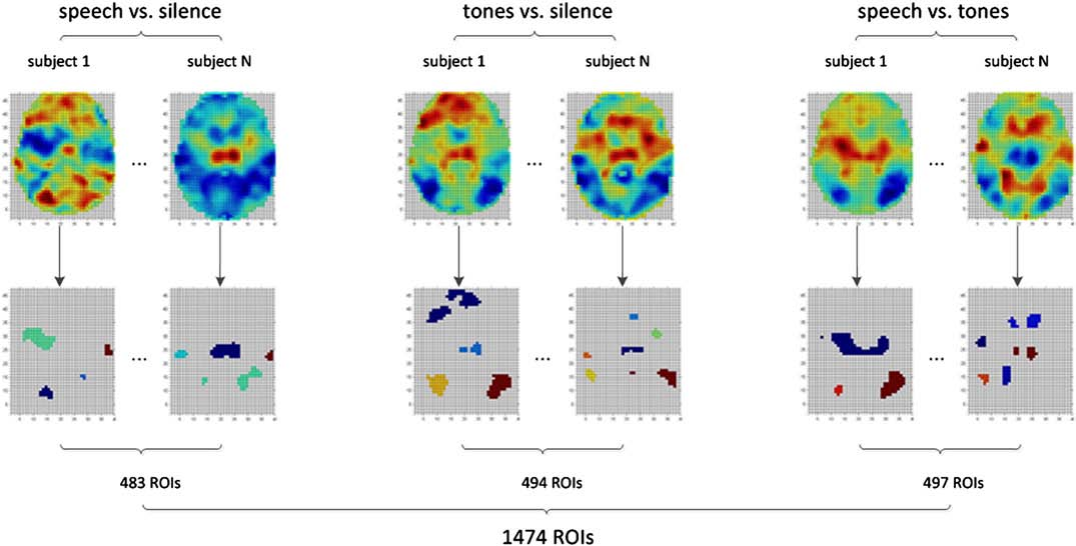
Vectorization of contrast maps. The first row of images is the original contrast maps, and the second row is the extracted ROIs. For visualization purposes, we show only one slice of a 3D brain image. For each type of contrast, we delineated ROIs from all 39 subjects to construct our dictionary. The dictionary size of the contrast speech vs. silence, tones vs. silence and speech vs. tones was 483, 494 and 497, respectively. These three dictionaries were applied to the corresponding contrast maps for each subject. As a result, each subject was represented with a 1474-D vector.

Normalized Z-maps were thresholded to select voxels with extreme contrast values for subsequent analysis. Among the selected voxels, we connected the voxels which were adjacent to each other in a 3D neighborhood, in which each voxel had 26 neighbors if it was not on the border. As a result, the selected voxels were merged into a set of disjoint regions, each of which was defined as a region of interest (ROI) (Dykstra, 1994; Pokrajac et al., 2005). To prevent mixing positive voxels and negative voxels in a single ROI, which could negate the signal, we considered these two categories of voxels separately. Positive voxels were ranked decreasingly whereas negative voxels were ranked increasingly according to their activation magnitudes. Only the top 5% of each category were selected. The cutoff of 5% was chosen because it outperformed other cutoffs, 1% and 10%, with respect to the classification performance. In this way, a number of ROIs were delineated to characterize the pattern of a contrast map. Due to individual differences and random noise, however, the set of ROIs delineated from different subjects varied significantly. To address this problem, we delineated a set of ROIs based on each subject, and applied all ROIs derived from all subjects to each single subject to form a long vector for each subject, with each dimension representing the mean contrast value over all voxels within the corresponding ROI. Finally, we concatenated the vectors from the three contrast maps, and obtained a 1474-dimension vector for each subject. In other words, each significantly activated/deactivated region was treated as a word, and all words occurring across all subjects constituted the dictionary. The frequency of each word was measured by the mean contrast value. An intuitive view of the contrast map vectorization process is shown in Fig. 3.

Since we performed ROI detection on each contrast map and then concatenated all the ROIs together, ROIs that were consistent among subjects were detected more than once. To merge those similar ROIs into one single feature, we performed a hierarchical clustering with average linkage (Johnson, 1967). The original space was represented as:

(9) $$\left[\begin{array}{ccc} S_{\left(1, 1\right)} & \cdots & S_{\left(1, 1474\right)}\\ \vdots & \ddots & \vdots \\ S_{\left(N,1\right)} & \cdots & S_{\left(N,1474\right)} \end{array}\right]$$

where each row represents a training sample and each column represents a ROI, *S*_(*i*,*j*)_ is the mean contrast value of ROI *j* for subject *i*, *N* is the total number of subjects. The distance between two ROIs was calculated as the Euclidean distance:

(10) $$\mathrm{dist}\left({\mathrm{ROI}}_{i},{\mathrm{ROI}}_{j}\right)=\sqrt{\sum_{k=1}^{N}{\left(S_{\left(k,i\right)}-S_{\left(k,j\right)}\right)}^{2}}$$

We cut the hierarchical tree with the inconsistency coefficient of 0.01, and calculated the mean value of the ROIs that were clustered together as the value of the joint feature. The cutoff of 0.01 was easily determined since the cluster results did not change in the cutoff range from 0.01 to 0.7. After hierarchical clustering, the dimensionality was reduced to 969.

#### 2.3.2 Sedation method

Subjects were sedated with three different sedation methods during the MRI scanning. Different sedation methods were expected to affect the activation pattern differently (DiFrancesco et al., in press). Therefore, we added sedation method as an additional feature, which was represented as a 3D binary vector

(11) $$\left[\begin{array}{ccc} 1 & 0 & 0\\ 0 & 1 & 0\\ 0 & 0 & 1 \end{array}\right].$$

As shown in the matrix defined in Eq. (11), each row of the matrix represented one of the three sedation methods. In this way, we represented each subject as a 972-dimension feature vector, including 969 features from the contrast maps after hierarchical clustering and 3 binary features from sedation method. Therefore, our dataset was represented as D defined in Eq. (12):

(12) $$D=\left\{\left(x^{\left(1\right)},y^{\left(1\right)}\right),\cdots ,\left(x^{\left(i\right)},y^{\left(i\right)}\right)\cdots ,\left(x^{\left(39\right)},y^{\left(39\right)}\right)|x^{\left(i\right)}\in R^{972}\right\}$$

where *x*^(*i*)^ and *y*^(*i*)^ was the feature vector and group label (NH or HI) for the *i*-th subject, respectively. This dataset D was used for subsequent feature selection and model learning.

#### 2.3.3 Feature selection and model learning

The WEKA software package was utilized to select a subset of features that were highly correlated with class labels and uncorrelated with each other (Hall, 1999). The merit of a subset of features was measured as:

(13) $$M_{S}=\frac{k\bar{r_{\mathrm{cf}}}}{\sqrt{k+k\left(k-1\right)\bar{r_{\mathrm{ff}}}}}$$

where $\bar{r_{\mathrm{cf}}}$ was the mean correlation between class label and selected features, $\bar{r_{\mathrm{ff}}}$ was the mean correlation between two features, *k* was the number of features in subset *S*. Greedy hill-climbing augmented with a backtracking facility was applied to search through the space of feature subsets (Dechter and Pearl, 1985). For explanation purposes, we can imagine that there was a rooted tree, which had included all possible feature subsets. In this tree, each node was a feature subset, which was represented as a 972 dimensional binary vector, with 1(0) indicating that the corresponding feature was (not) selected. Each node had 972 successors/children, each of which was generated by flipping one of the 972 dimensions of the current node. Our goal was to step through this tree to find a node with relatively high *M_s_*. In practice, the whole tree would not be constructed because it was unlimited. Only the successors were generated whenever needed. The search started from the root, which was the empty set of features in our project, and repeatedly chose the successor with the highest *M_s_* at each node. The search terminated when 5 consecutive non-improving steps occurred. With the selected subset of features, we trained a linear SVM classifier (Chang and Lin, 2011).

#### 2.3.4 Predicting new subjects

Given a new subject, we first normalized the contrast maps to the infant template space (Altaye et al., 2008), so that the given contrast maps were registered with the training contrast maps. A 972-D feature vector was then constructed with procedures described above, which was subsequently filtered based on the feature selection results obtained from the training set. Finally, the formatted feature vector was fed to the trained classifier, yielding a decision score (fMRI_score) for the new subject based on the functional MRI data alone. The rule for classification was formulated as:

(14) $$\mathrm{Class}\;\mathrm{label}=\left\{\begin{array}{cc} \mathrm{HI} & ,\mathrm{if}\;\mathrm{fMRI}\_\mathrm{score}\geq \epsilon_{f}\\ \mathrm{NH} & ,\mathrm{otherwise} \end{array}\right).$$

#### 2.3.5 Important features

The importance of a feature was measured as follows:

(15) $$I\left(f\right)=\left|\sum_{i=1}^{N}\sigma_{i}w_{\mathrm{if}}\right|$$

where || was the absolute value function, *N* was the total number of folds of cross-validation as described in the following part, *w_if_* was the SVM weight for feature *f* during *i*-th fold of cross-validation, *σ_i_* = 1 if the feature *f* was selected in the *i*-th fold of cross-validation. Otherwise, *σ_i_* = 0. For the ROIs that were merged into a joint feature through the hierarchical clustering, the importance of such an ROI was equal to the importance of the feature, to which this ROI belonged.

### 2.4 Integrated model

**Fig. 4:**
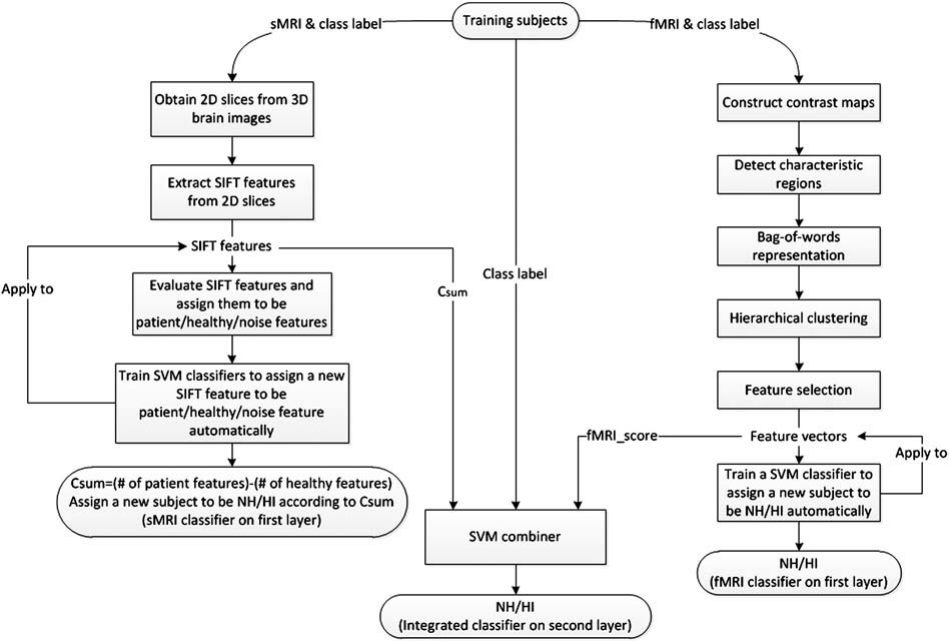
Framework for the two-layer classifier.

To combine the sMRI and fMRI data, we designed a two-layer classification model (Fig. 4). Given a training set, we trained two classifiers, namely sMRI classifier and fMRI classifier. Then we applied these two classifiers to the training set. As a result, we obtained two predicted scores for each training sample. Thus, the original feature space was transformed into a new two-dimensional feature space through these two classifiers. Finally, we trained a linear SVM classifier (with parameter C = 1) in the new feature space to combine the two scores together.

When predicting new subjects, we first obtained the two predicted scores from the sMRI classifier and fMRI classifier, then fed these two predicted scores into the second layer classifier to yield the final decision score *y*. The decision rule was defined as follows:

(16) $$y=f\left(C_{\mathrm{sum}},\mathrm{fMRI}\_\mathrm{score}\right)=w_{1}*C_{\mathrm{sum}}+w_{2}*\mathrm{fMRI}\_\mathrm{score}+\mathrm{bias}$$

(17) $$\mathrm{Class}\;\mathrm{label}=\left\{\begin{array}{cc} \mathrm{HI} & ,\mathrm{if}\;y\geq \epsilon_{i}\\ \mathrm{NH} & ,\mathrm{otherwise} \end{array}\right)$$

where *w*_1_, *w*_2_ and *bias* were the parameters in the SVM model, which were learnt from the training.

### 2.5 Validation of the classifier

Leave-one-out cross-validation (LOOCV) was employed to validate the three classifiers as follows. The total number of subjects was denoted as *N*. We performed *N* experiments, each of which was called one fold of cross-validation. In the *n*-th (n = 1,…,N) fold of cross-validation, the *n*-th subject was used for testing; while the others were used for training. Threshold *ϵ*_*s*_ was determined so that the false positive rate and false negative rate for the training brains were equal, while *ϵ*_*f*_ and *ϵ*_*i*_ were set to be 0. These thresholds were applied to the test images to assign them to be either NH or HI. The classification accuracy for all the *N* subjects was reported as accuracy. Equal error rate (EER) accuracies were also determined based purely on the predicted scores of the testing brain images, e.g. the threshold *ϵ*_*s*_/*ϵ*_*f*_/*ϵ*_*i*_ were chosen so that the false positive rate was equal to false negative rate for the testing brains. In addition, area under curve (AUC) was also calculated to evaluate the performance of classifiers.

## 3 Results

### 3.1 Classifier performance

**Fig. 5:**
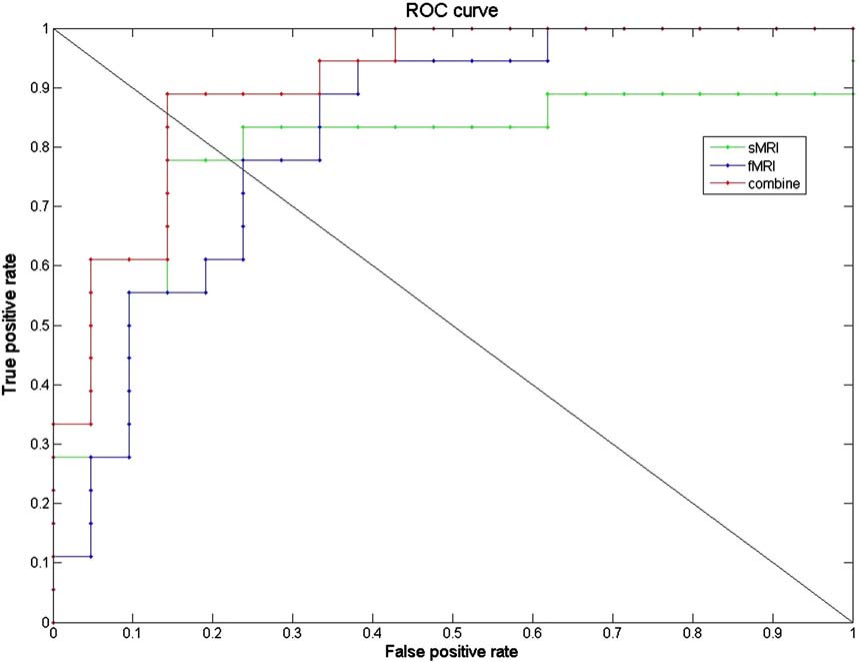
ROCs of the three classifiers.

**Fig. 6:**
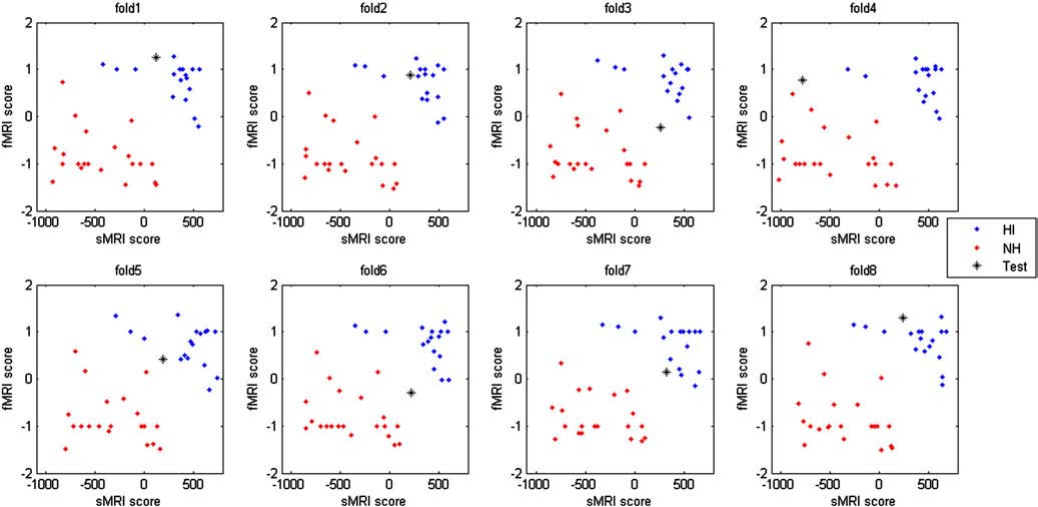
Distribution of sMRI–fMRI scores. Each panel is one-fold of cross-validation. The horizontal axis is the output of the sMRI classifier and the vertical axis is the output of the fMRI classifier. Blue dots are HI training samples, red dots are NH training samples, the black star is the testing sample. The true label of the testing sample is HI for all the 8 folds of cross validation. Figures for all the 39 folds of cross-validation can be found in Fig. S1. (For interpretation of the references to colors in this figure legend, the reader is referred to the web version of this article.)

Performances of the three classifiers are shown in Table 1, and receiver operating curves (ROCs) are plotted in Fig. 5. While the sMRI classifier and fMRI classifier performed well individually, their combination achieved a significant improvement in performance. The combined classifier yielded AUC and EER as high as 0.90 and 0.89, respectively. From the ROC, we can see that the sMRI classifier could not predict some of the positive subjects (HI) correctly even when the decision threshold was set to be very low, because the classifier did not reach 100% true positive rate even when the false positive rate approached 100%. However, the ROC for fMRI was in an opposite situation. The ROC did not reach 0% false positive rate even when the true positive rate approached 0%, suggesting that the fMRI classifier had difficulty in classifying some of the negative subjects (NH) correctly. As sMRI and fMRI classifiers were vulnerable to different types of errors, it was possible to combine them to overcome their individual limitations. To illustrate the reason why the combination can be successful, we plotted sMRI–fMRI scores in Figs. 6 and S1. Simply speaking, the fMRI classifier draws a horizontal line to separate the two groups of subjects based on the fMRI data, while the sMRI classifier draws a vertical line to separate the two groups based on the sMRI data. Obviously, the two groups could not be perfectly separated by either a horizontal or a vertical line in Figs. 6 and S1. However, by combining the fMRI and sMRI classifiers, the two groups of subjects were separable with a diagonal line as shown in the figures.

Performances of the three classifiers are shown in Table 1, and receiver operating curves (ROCs) are plotted in Fig. 5. While the sMRI classifier and fMRI classifier performed well individually, their combination achieved a significant improvement in performance. The combined classifier yielded AUC and EER as high as 0.90 and 0.89, respectively. From the ROC, we can see that the sMRI classifier could not predict some of the positive subjects (HI) correctly even when the decision threshold was set to be very low, because the classifier did not reach 100% true positive rate even when the false positive rate approached 100%. However, the ROC for fMRI was in an opposite situation. The ROC did not reach 0% false positive rate even when the true positive rate approached 0%, suggesting that the fMRI classifier had difficulty in classifying some of the negative subjects (NH) correctly. As sMRI and fMRI classifiers were vulnerable to different types of errors, it was possible to combine them to overcome their individual limitations. To illustrate the reason why the combination can be successful, we plotted sMRI–fMRI scores in Fig. 6 and S1. Simply speaking, the fMRI classifier draws a horizontal line to separate the two groups of subjects based on the fMRI data, while the sMRI classifier draws a vertical line to separate the two groups based on the sMRI data. Obviously, the two groups could not be perfectly separated by either a horizontal or a vertical line in Fig. 6 and S1. However, by combining the fMRI and sMRI classifiers, the two groups of subjects were separable with a diagonal line as shown in the figures.

### 3.2 Feature selection in sMRI analysis

In the analysis of sMRI data, image features were selected based on their likelihood scores. The total number of image features in a brain image ranged from 35,000 to 52,000. Most of these image features were noise features. The total number of selected features, i.e., healthy and patient features, ranged from 300 to 1400 for different brains with a likelihood threshold of 0.9. Different choices of likelihood threshold for the sMRI feature selection resulted in different numbers of selected features and therefore different classification accuracies. Table 2 shows the relation between classification accuracy and the likelihood threshold. The classification accuracy did not change for likelihood threshold ranging from 0.7 to 1.1. The AUC changed within a range of 0.09 with a peak where the likelihood threshold equaled 0.9. The EER accuracy varied within a range of 0.08. All three classification performance measures were stable with different likelihood thresholds.

### 3.3 Stability of feature selection in fMRI analysis

We have analyzed the stability of feature selection in the analysis of fMRI data. There were in total 972 features as the input for feature selection. Only 6.2% of the features (with a total number of 60) were selected at least once. For each fold of cross-validation, there were usually about 20 features selected for the training, generally 30% of which were consistently present in all folds of cross-validation. We calculated a stability index as follows (Kalousis et al., 2007):

(18) $$\mathrm{Sim}\left(s_{i},s_{j}\right)=\frac{\left|s_{i}\cap s_{j}\right|}{\left|s_{i}\cup s_{j}\right|}$$

(19) $$\mathrm{index}=\frac{2}{c\left(c-1\right)}\sum_{i=1}^{c-1}\sum_{j=\left(i+1\right)}^{c}\mathrm{Sim}\left(s_{i},s_{j}\right)$$

where *c* was the total number of rounds of feature selection, *s_i_* and *s_j_* were two sets of features selected during two runs, |*s*_*i*_∩*s*_*j*_| was the cardinality of the intersection between *s_i_* and *s_j_*, and |*s*_*i*_∪*s*_*j*_| was the cardinality of the union of *s_i_* and *s_j_*. Our feature selection yielded a stability index of 66.2%, which indicated that 66.2% of the selected features, on average, were common between any two runs of feature selection. Since the Euclidean distance was used in the hierarchical clustering, only very similar ROIs were merged. There was still considerable redundancy among features. For example, two ROIs, e.g. one from the contrast speech vs. silence and the other from the contrast tones vs. silence, were significantly correlated with class labels, and meanwhile they were also highly correlated with each other. Due to the large Euclidean distance between them, however, they were not merged during the hierarchical clustering. In feature selection, these two ROIs were treated as different features and selected interchangeably. This caused the calculated stability index to be lower than the actual value. In this regard, 66.2% represented very high stability.

### 3.4 Discriminative brain regions

For sMRI, we measured the importance of a SIFT feature with its likelihood score. In our project, however, the SIFT features usually had a scale of 10 mm or even larger, and correspondingly the side length of the appearance matrices was larger than 40 mm. Due to the large size of the SIFT features, it was more difficult and less useful to interpret the medical implications of such large brain regions.

**Fig. 7:**
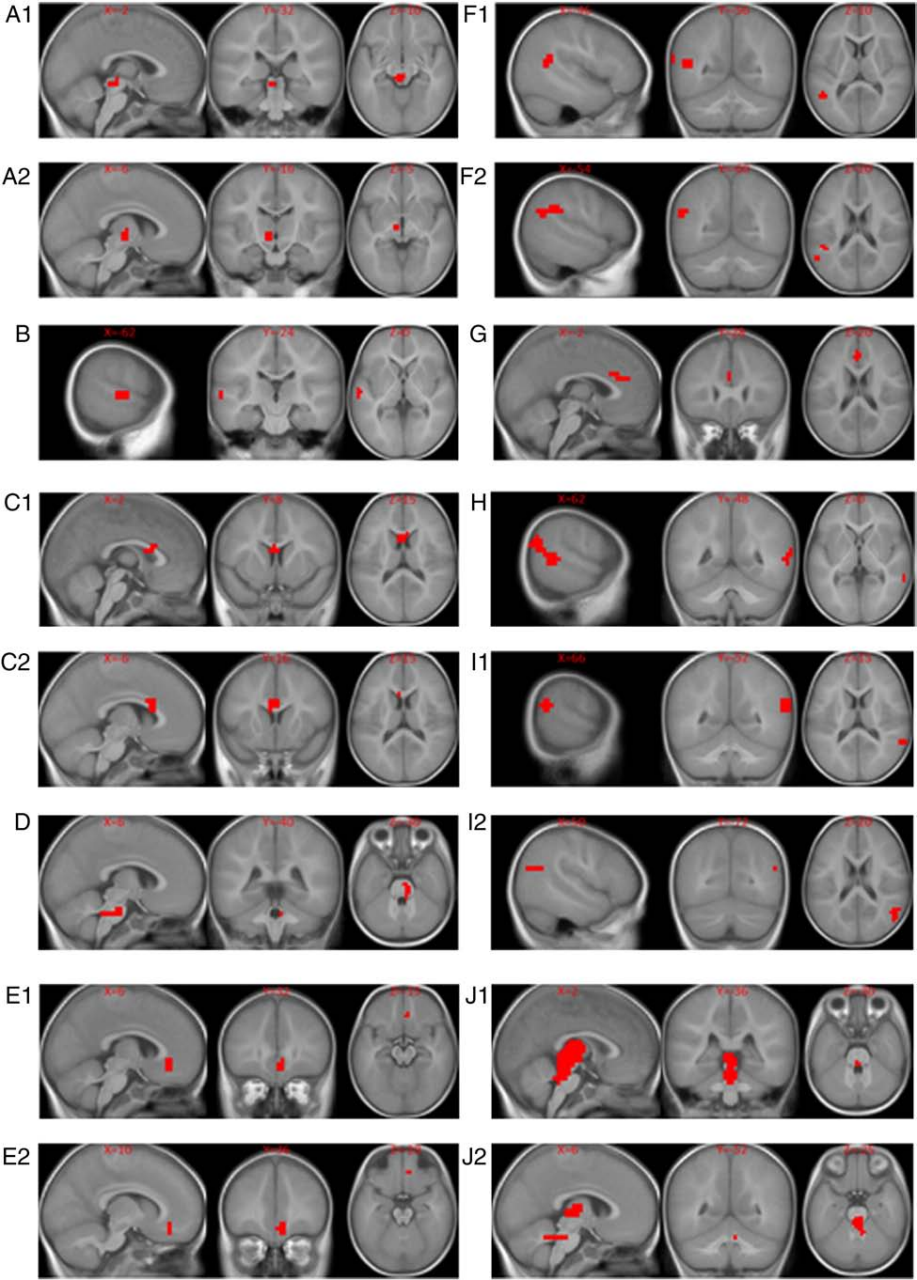
Important brain regions identified by the fMRI classifier as differentiating the hearing impaired group (HI) from the normal hearing control group (NH). The brain regions are visualized with the xjview toolbox (http://www.alivelearn.net/xjview). Images are displayed in neurological orientation.

With those considerations, we only focused on the highly predictive brain regions identified by the fMRI classifier. Fig. 7 shows the top 10 functional features extracted from fMRI data that differentiate the HI and NH groups. Features are numbered from A to J in order. ROI A1 and A2 were merged during hierarchical clustering into a joint feature A. Similar procedures were performed for features C, E, F, I and J. We can see that ROIs grouped together during hierarchical clustering are always from the same type of contrast maps (Table 3) and encompass adjoining or sometimes overlapping brain regions as designated by Brodmann's Areas in the 4th column of Table 3.

## 4 Discussion

In this work, we have built a robust two-layer classifier that can accurately separate HI from NH infants. We realize that hearing in newborns can be accurately tested using the auditory brainstem response (ABR) evaluations or the otoacoustic emission (OAE) measures, it is thus not our intention to develop a tool for computer-aided diagnosis of hearing loss. Rather we provide a proof of principle that it is possible to accurately determine the functional, developmental status of the central auditory system in congenitally hearing impaired children based on MR images alone by utilizing machine learning techniques. Such success has been previously reported in other progressive diseases, such as Alzheimer's disease (Cuingnet et al., 2011). However, for many progressive diseases, definite diagnosis is often difficult to establish, in which case the LOOCV approach may not be able to estimate the classifier performance accurately. Therefore, our dataset with solid labels corresponding to diagnostic categories of the participants that have NH or HI enables us to make an objective evaluation of our algorithm, and demonstrate conclusively the feasibility of using machine learning in making automated diagnoses or prognoses based on imaging examinations. The approach described here may not be limited to a specific disease; essentially, any disease dataset with sMRI and fMRI brain images can be analyzed with our method provided that sufficient training data is available.

A major innovation that makes highly accurate predictions possible in our approach is that we extracted high-level features instead of using each single voxel as a feature as in traditional approaches. The SIFT features from sMRI images and region-level features from fMRI images are much less sensitive to registration errors when compared to voxel-features. In addition, utilization of high-level features can considerably reduce the dimensionality of feature space, which not only makes our classification problem easier to handle, but also helps to reduce the problem of over-fitting. At last, our classification model is more interpretable, because our model involves fewer features consisting of continuous regions instead of scattered voxels. These features can then be related more easily to disease etiology, diagnosis and prognosis.

Another innovation of our approach is that we employed a bag-of-words strategy to analyze the functional contrast maps. This technique can characterize the activation pattern for every individual in spite of the great variability in the activation pattern among individuals. Considering the relatively small sample size, we constructed our feature pool with all available samples, including the one used for testing during the cross-validation. We implemented a variant version of our algorithm, in which we extracted ROIs based only on the training samples, and subsequently applied those ROIs to the testing sample directly. As expected, the variant algorithm performed slightly worse (AUC = 0.81) than our original algorithm (AUC = 0.83). Adding the ROIs from new samples requires us to retrain the classifier every time when new samples are available. As the feature pool becomes larger in the future, the retraining is not necessary.

Integration of different types of data, e.g. data from multiple modalities, has been demonstrated to be more powerful for classification (Fan et al., 2007, 2008; Tosun et al., 2010; Wang et al., 2012). However, how to implement such integrations in the best way remains to be explored (Orru et al., 2012). Traditionally, features from different types of data are concatenated and a single classifier is trained (Fan et al., 2007, 2008; Tosun et al., 2010; Wang et al., 2012). Specifically, the traditional integration method requires the training set to be organized into matrices, with each row representing a training sample and each column representing a feature. One matrix is constructed for one type of data, and subsequently all the matrices are concatenated into one big matrix, which serves as the input for classifier training. In our project, the fMRI data can be easily organized in this way. For sMRI, however, each training sample has a set of SIFT features, which can be treated as a set of words included in an article. Different articles have different sets of words. Thus, it is not easy to organize the sMRI data into a matrix as described above, and the traditional integration method is not applicable. Under such circumstances, we proposed a two-layer model to integrate the sMRI and fMRI data. Since the traditional approach was not applicable in our project, we did not compare their performances in the present paper. Additionally, our two-layer model is also applicable when features from different modalities can be concatenated. In this case, one classifier is trained for one modality, and a second-layer classifier is subsequently used to integrate the multiple classifiers on the first-layer. This approach is able to combine as many types of data as possible, without worrying about the high dimensionality or overfitting.

Although computer-aided diagnosis of hearing loss is not needed, our algorithm can potentially advance the study of congenital hearing loss mechanism by identifying discriminative brain regions as disease biomarkers for hearing impairment at various levels in the auditory system. Inspecting the most important features that differentiate children born with hearing impairment from children with normal hearing in this study, we see some features that are in line with hypotheses about under stimulation of auditory function in HI infants; while other observations already begin to add to our knowledge of how congenital deafness affects brain development and function. For example, features B, F, H, and I include known components of the auditory language network which our group and others have previously shown to be engaged by the narrative comprehension task (Karunanayaka et al., 2007; Schmithorst et al., 2006). These features include (B) the planum temporale and primary auditory cortex in the left hemisphere (including Wernicke's area, the classical language recognition module), as well as the angular gyrus and supramarginal gyrus at the temporal parietal junction of the (F) left and (H, I) right hemispheres, known auditory and visual language association regions. Although all participants were bilaterally severely to profoundly hearing impaired, we observe left dominant auditory/language related activity present in components A, B, and F. In addition, components H and I contain right hemisphere auditory/language activity. Functional features such as these are not unexpected in terms of regions of differential cortical activation between HI and NH children listening to natural language as an auditory stimulus and it is reassuring to see these regions highlighted by our algorithm as potential biomarkers corresponding to hearing impairment. Similarly, there is evidence of differential activation in subcortical features corresponding to the auditory brainstem pathways. Features A, D, and J include elements of the reticular auditory pathway of the brainstem which has been identified by electrophysiological studies to have a key role in auditory perception of location of sounds as well as the ability to filter a source of sound in background noise. Roughly these features appear to encompass key elements of the auditory pathway at the level of the pons (D) including the cochlear nucleus, trapezoid body, lateral lemniscus and superior olive on the right, (A) inferior colliculus, medial geniculate on the left and (J) thalamus bilaterally (Kretschmann and Weinrich, 1998). Although the resolution of the fMRI scans (4 × 4 × 5 mm) is not sufficient to resolve these structures individually, differences in activation in these regions, as indicated by reference to the higher resolution anatomical images, suggest that brain stem auditory nuclei may be involved.

One feature that is conspicuously absent from those illustrated in Fig. 7 is the primary auditory cortex (BA41). We expected that this region would be important in differentiating HI from NH participants and hoped that it could potentially become a biomarker for predicting outcome for hearing and language following cochlear implantation in HI infants as suggested by our earlier work (Patel et al., 2007). The sedation used in the present study is a likely confounding to primary auditory function and may be partly responsible for the absence of a functional MRI feature in primary auditory cortex that differentiates the groups (DiFrancesco et al., in press). However, because Fig. 7 highlights differences between the groups that optimally separate them, it is possible that brain regions beyond primary auditory cortex that are responsible for recognizing sounds as speech and for extracting and associating content are more differentially stimulated in a scenario where the hearing impaired brain receives a rare auditory input that is above the threshold it can detect. Vibrations, loud noise and other stimuli may occasionally stimulate the auditory cortex in a deaf infant so that it is capable of processing sound and responds during our experiment in the same manner as the NH children who are receiving sound stimulation at the same relative SPL. However, unless the HI infant is participating in a successful hearing aid trial, it is much less likely that they are routinely subjected to an auditory stream of speech that is consistently above their hearing threshold and hence unintelligible. HI infants in this study were all severe to profoundly hearing-impaired and ultimately received a cochlear implant because they did not derive sufficient benefit from an external hearing aid. Though this explanation is speculative, it could explain why features B, C, E, F, G, H, and I seem to be more important in separating the HI and NH groups of infants based on brain activation during fMRI.

On the other hand, our analysis on the fMRI data in this study also identified a number of areas that are not necessarily expected to play a role in differentiating HI from NH children. In particular, several functional features also appear in various portions of the anterior cingulate cortex (ACC, BA 24,32,33): areas associated with attention management, conflict monitoring, and error detection (Weissman et al., 2005). These features may be related to responses in the HI group to the novel auditory stimulus. ACC features are present in all three contrasts (C2, E1, E2, and G), suggesting a difference in response to sound input in the HI group who do not typically receive an auditory input at a level above their auditory threshold. Important features are also present in secondary visual cortex (H) (BA18), associative visual cortex (BA19) and other subcortical regions; differentiating the two groups. These features provide clues about additional neuroimaging biomarkers that may be relevant to the future use of functional neuroimaging to guide predictions about speech and language outcomes in HI infants who receive a cochlear implant. This type of prognostic information, currently not available, is obviously of great significance. For example, it helps to calibrate the expectations and avoid subsequent disappointment, save money of the family and avoid anesthetic risks when it is clear that a child will derive no benefit from the procedure.

In the present study, all infants were sedated for a clinical MRI scan and the fMRI task was appended to the end of the protocol. Further, there were different agents used for the sedation in the population we sampled, including propofol, Nembutal and sevoflurane. These drugs may have a different influence on the BOLD signal we detected. Note that the influence of sedation is to attenuate the auditory and language related brain activity and corresponding BOLD signal relative to what would be detected in awake or even sleeping babies (Difrancesco et al., 2011; DiFrancesco et al., 2013, in press; Wilke et al., 2003). Therefore, the current approach for automatic classification of NH vs. HI would likely be more effective in a scenario where fMRI data could be recorded from the participants without the influence of sedation. Demonstrating that our approach can accurately classify infants by hearing status even under the confounding influence of sedation encourages optimism for other applications where confounding disease-related conditions may modify the BOLD signal, such as cerebrovascular diseases.

In the future, we will try image segmentation algorithms to define ROIs instead of thresholding the contrast maps. Other evidence, such as tissue density maps and functional connectivity networks, may be integrated into our model. For example, we can train a classifier based on the tissue density maps and then integrate it into our model with the second-layer classifier. Beyond the MRI data, our model will also permit integration from electrophysiologic imaging modalities such as evoked response potentials (ERP), electroencephalography (EEG), or magnetoencephalography (MEG). These brain scanning techniques directly record brain activities; however they are limited in their spatial resolution by the algorithms that are used to localize sources of brain activity based on recordings at the surface of the skull. Combining MR imaging features with electrophysiologic features recorded directly from brain responses to auditory input could leverage the benefits of each imaging modality to produce much more accurate predictions about patient outcomes. Due to the inherent properties of our two-layer model, integration of other evidences can be easily implemented. With the improved classifier, the method is likely to have applications to many other diseases.

## 5 Conclusion

First, our study demonstrates that HI and NH infants can be differentiated by brain MR images, e.g. different fMRI contrasts in auditory language network and auditory brain stem nuclei. Based upon the discriminative features, a classification model can be built to predict whether an individual has normal hearing or impaired hearing. The discriminative features may also be used as objective biomarkers of hearing loss or used for further disease mechanism studies. Secondly, our two-layer model integrates sMRI and fMRI in an effective way. While our sMRI classifier and fMRI classifier work moderately well individually, the combination of the two classifiers gives birth to a much more powerful classifier, which corroborates the hypothesis that integration of multiple modalities improves classification accuracy. Besides, our integration approach is very flexible, and it can be easily extended to include many diverse types of data. Future work with this machine learning approach to automated image classification may allow us to make predictions about speech and language outcomes in individual children who receive cochlear implants for remediation of congenital hearing impairment.

The following are the supplementary data related to this article

Fig. S1 Distribution of sMRI–fMRI scores for all 39 folds of cross validation. Each panel is one-fold of cross-validation. Horizontal axis is the output of the sMRI classifier and vertical axis is the output of the fMRI classifier. Blue dots are HI training samples, red dots are NH training samples, the black star is the testing sample. The true label of the testing sample is HI for fold1 to fold18, and NH for fold19 to fold39.

Supplementary data to this article can be found online at http://dx.doi.org/10.1016/j.nicl.2013.09.008.

## Figures and Tables

**Table 1:** Classification performance of the three classifiers.

	Specificity	Sensitivity	Accuracy	AUC	EER
sMRI	0.83	0.62	0.72	0.78	0.78
fMRI	0.76	0.72	0.74	0.83	0.76
sMRI + fMRI	0.86	0.89	0.87	0.90	0.89

**Table 2:** Classification performance with different likelihood thresholds for feature selection from sMRI data.

Likelihood threshold	0.7	0.8	0.9	1.0	1.1
Accuracy	0.72	0.72	0.72	0.72	0.72
AUC	0.69	0.71	0.78	0.70	0.74
EER	0.69	0.69	0.77	0.77	0.77

**Table 3:** Characteristics of the top functional ROIs. STG is short for singular temporal gyrus, MTG for middle temporal gyrus.

Feature	Number of voxels	Contrasts	BA areas	Anatomical labels
A1	16	Speech vs. silence	Red Nuc.	Red Nuc.
A2	10	Speech vs. silence	Thal., Sub Thal. Nuc	Thal., Sub Thal. Nuc.
B	19	Speech vs. tones	22	STG
C1	19	Tones vs. silence	33	Pregenual Cing. G.
C2	15	Tones vs. silence	33,24	Pregenual Cing. G., Vent. Ant. Cing. G.
D	14	Tones vs. silence	Auditory nuclei	Pontine Auditory Nuclei
E1	12	Speech vs. tones	32	Cing. G.
E2	11	Speech vs. tones	32,10	Cing. G., Prefrontal Cortex
F1	60	Tones vs. silence	22,40,39,13	STG, Super Marg. G, Angu. G., Ins.
F2	40	Tones vs. silence	40,39,13	Super Marg. G, Angu. G., Ins.
G	11	Speech vs. silence	24,32	Vent. Ant. Cing. G.,Cing. G.
H	115	Speech vs. silence	19,22,39,18,21,40	Mid Occ. G., STG, Angu. G., Lingual G, MTG, Super Marg. G
I1	24	Speech vs. silence	22,40,39	STG, Super Marg. G, Angu. G.
I2	30	Speech vs. silence	40,39	Super Marg. G, Angu. G.
J1	253	Tones vs. silence	Thal., Red Nuc.	Thal., Red Nuc.
J2	142	Tones vs. silence	Thal., Mamillary Body	Thal., Mamillary Body
