# Supplementary material for: Combined analysis of sMRI and fMRI imaging data provides accurate disease markers for hearing impairment
Source: Neuroimage Clin. 2013 Oct 11;3:416–28. doi: 10.1016/j.nicl.2013.09.008 (PMC3863984; doi:10.1016/j.nicl.2013.09.008)
Supplement: Fig. S1 — Distribution of sMRI–fMRI scores for all 39 folds of cross validation. Each panel is one-fold of cross-validation. Horizontal axis is the output of the sMRI classifier and vertical axis is the output of the fMRI classifier. Blue dots are HI training samples, red dots are NH training samples, the black star is the testing sample. The true label of the testing sample is HI for fold1 to fold18, and NH for fold19 to fold39. [file mmc1.pdf]

Supplementary Materials

Fig. S1. Distribution of sMRI-fMRI scores for all 39 folds of cross validation. Each panel is one-fold of cross-validation. Horizonatal axis is the output of sMRI classifier and vertical axis is the output of fMRI classifier. Blue dots are HI training samples, red dots are NH training samples, the black star is the testing sample. The true label of the testing sample is HI for fold1 to fold18, and NH for fold19 to fold39.

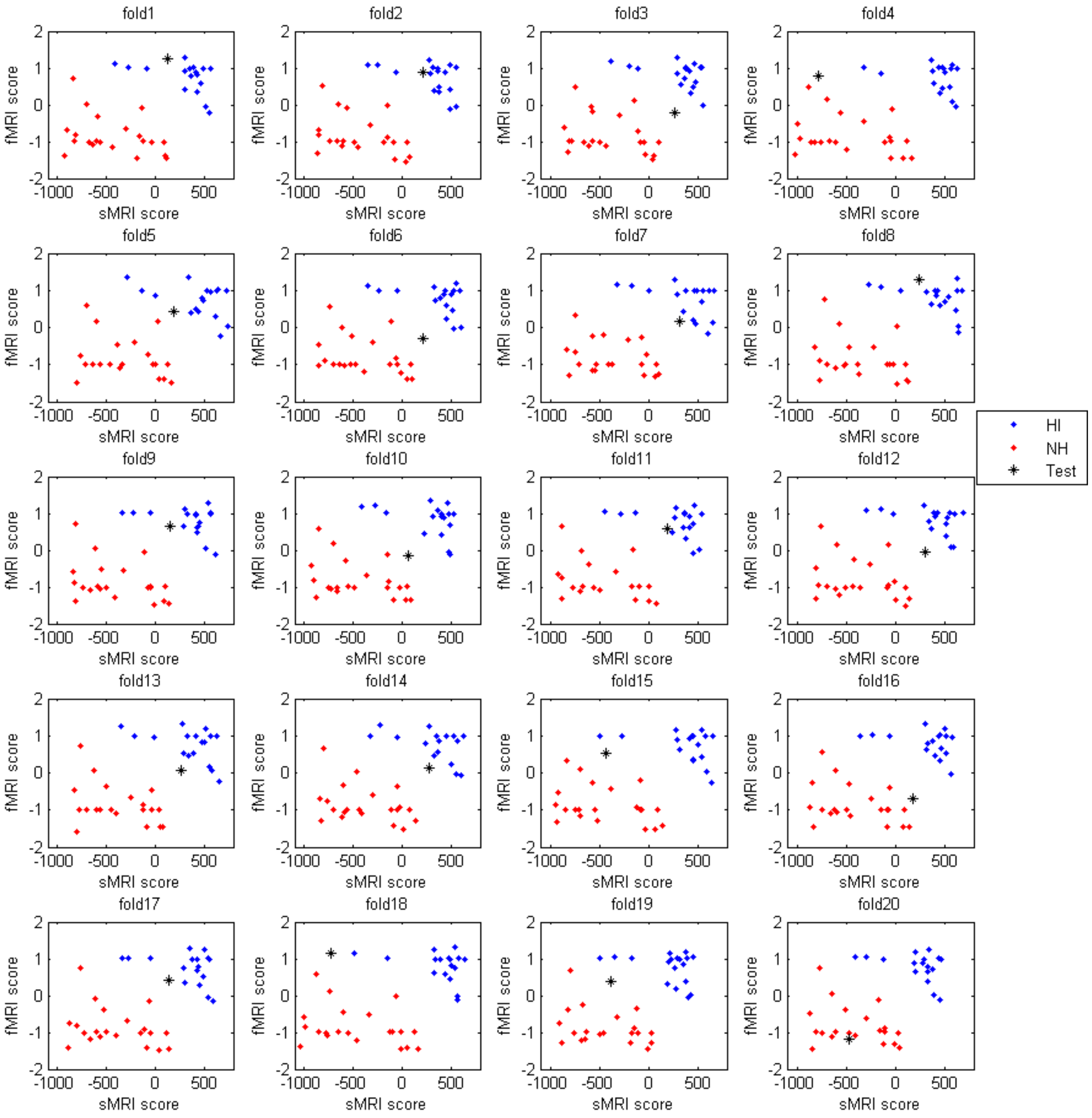

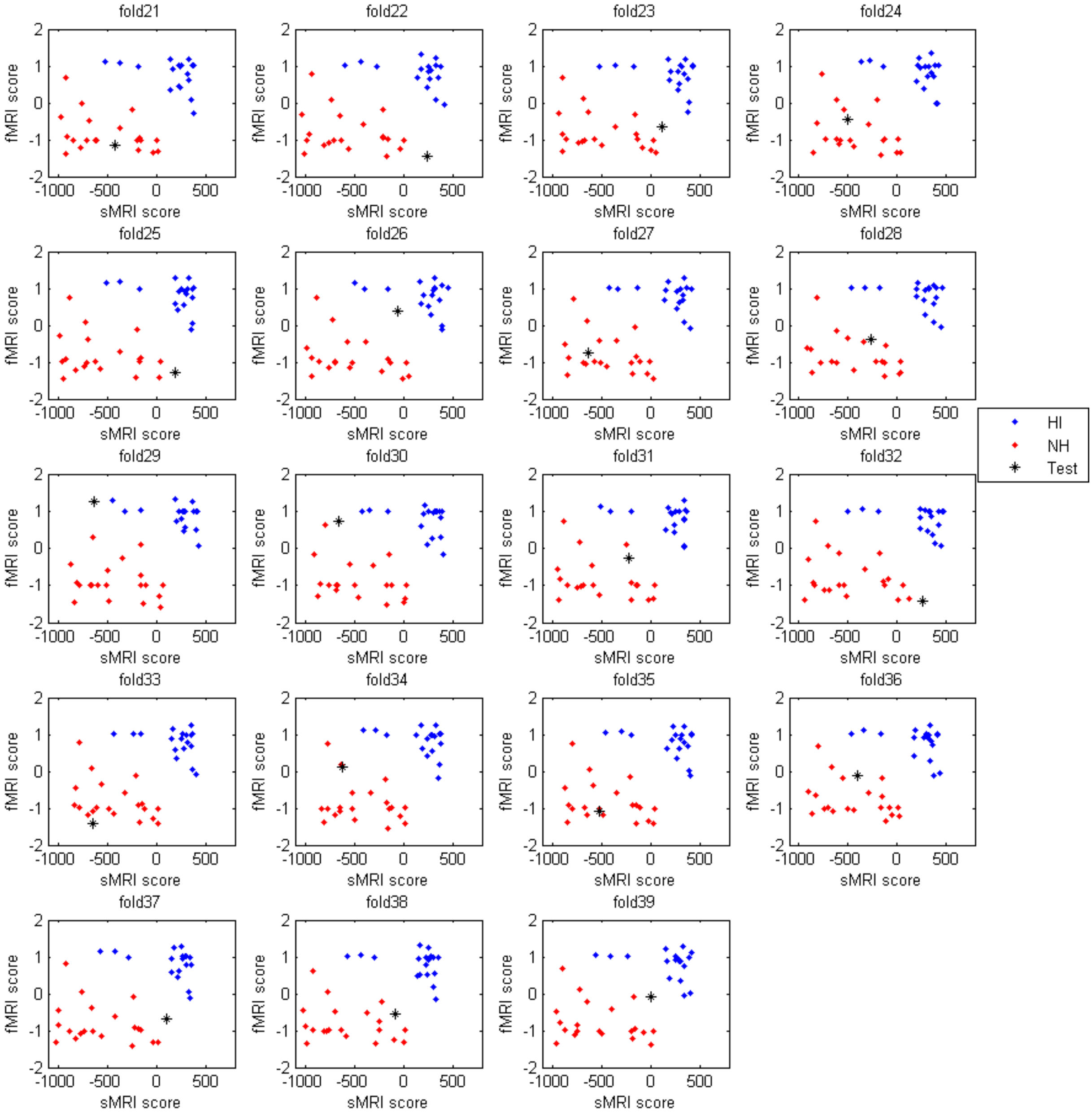
fold21
fold22
fold23
fold24
fold25
fold26
fold27
fold28
fold29
fold30
fold31
fold32
fold33
fold34
fold35
fold36
fold37
fold38
fold39
fMRI score
sMRI score
HI
NH
Test
